# Ham Green Hospital Extensions

**Published:** 1937

**Authors:** W. W. Jameson

**Affiliations:** Professor of Public Health, University of London; Dean of the London School of Hygiene and Tropical Medicine


### HAM GREEN HOSPITAL EXTENSIONS.

### THE OPENING ADDRESS DELIVERED

ON SEPTEMBER 23RD, 1937,

BY

Professor W. W. JAMESON, M.D., F.R.C.P., D.P.H., Barrister-at-Law,

*Professor of Public Health, University of London; Dean of the London School of Hygiene and Tropical Medicine.*

---

A VISIT to Bristol is for me always a delightful experience, even when the pleasure is somewhat tempered by the responsibility of having to talk for a short time to such a distinguished audience as that I see before me to-day. When I come to Bristol I feel I am entering a city with all the qualities of an old capital of the west country. Its history goes back into the distant past, and the story of its local government is so interesting that it provides teachers like myself with much material for lectures on the growth of public health administration in this country. While its early history repays investigation, its recent achievements are no less remarkable, and I believe there is no other city in the land where modern progressive local government may be studied with greater profit.

There are many directions in which you have given a lead in the field of public health: in the manner in which both University and City Council have united in their efforts to provide adequate public health services for Bristol; in your great programme of housing, with the gradual replacement of slums by modern housing estates complete with community centres; in the development of a scheme of health centres throughout the city, centres in which both general public health work, including the work of the sanitary inspectors, and school medical work are carried on in the service of preventive medicine.

Nor have your hospitals been neglected, as to-day's ceremony affords ample evidence. I understand that a large sum of money is being expended on the enlargement and modernization of the municipal hospital, and that considerable additional accommodation is being provided for maternity cases—a wise provision, as I am satisfied that more and more women will wish to be confined elsewhere than in their own homes. And then we have this hospital where we meet to-day—a hospital which has always been something more than merely up-to-date. The new pavilion for tuberculous cases is one of the most attractive and best planned of any I have seen, and the extensions, thanks to your architect, Mr. G. C. Lawrence, are more modern in their conception than any other isolation accommodation in the country. Not only are you providing small wards for patients who for their own sakes or in the interests of other patients should be isolated, you are doing what has not so far been done in any other hospital in England—arranging for the admission of cases of smallpox to a pavilion within the grounds of the isolation hospital, and administered by the medical superintendent of the whole institution. I have long hoped that such a scheme would be adopted by a local authority, and I am glad Bristol is leading the way. Such a step could not have been taken were you not fortunate in having in Dr. Peters a medical superintendent who is not only trusted in his own city, but is recognized by all public health workers as one of our leading authorities on infectious disease.

There are few cities able to show a record of progressive public health administration comparable with yours, and when foreign visitors come to me, as they do in considerable numbers, to ask where they should go to see a good example of English local government, I invariably direct them to the Capital of the West Country.

Though we have had general hospitals in England for a great many years, the specialized institution for the admission of cases of infectious diseases dates, for all practical purposes, only from the last century.* Prior to the coming of the fever hospital the old parish "pest-house," as it was called, was the only special provision for the treatment of the infectious sick. The first fever hospitals were sometimes known as "houses of recovery," as their original design was to promote the cure of the individual fever patient. Soon these hospitals began to be advocated on the ground of their protecting the household against the spread of infection.

It is noteworthy that smallpox had always been regarded as requiring a separate building to itself, but in the first fever hospitals all sorts of infectious cases were admitted indiscriminately to the wards. As a result doctors and nurses caught "fever" of one kind or another, while convalescent patients had what appeared to be recurrences of "fever" attacks. So serious did this situation become that people began to ask themselves whether special fever hospitals were really satisfactory, and to wonder whether fever cases

* The London Fever Hospital was founded in 1801.

should not rather be distributed throughout the wards of general hospitals. The experiment was indeed tried, but with disastrous results to the other patients and to the hospital attendants.

Gradually, however, doctors began to learn how to differentiate one type of infectious disease from another, and so it became possible to classify cases under separate headings and to arrange for cases of scarlet fever to be isolated in one set of wards, typhoid fever in another, typhus fever in yet another, and so on.

A well-known Local Government Board Report in 1882 pointed out that "even with fair separation between the several infections there remained a risk, which if small was still appreciable, that patients in these hospitals might contract fresh disease, and that instances were not wanting to show that the risk to patients, and to attendants as well, varied according to the goodness or badness of the arrangements of construction and administration of the hospital."

What was written over fifty years ago holds good to-day, and we know now that no fever hospital can be administered satisfactorily unless adequate isolation accommodation in one and two bed wards forms an important part of the ward provision of the institution. In proof of this I need only remind you that last year, apart from cases admitted for observation and cases of mixed infection, no fewer than seventeen different kinds of infectious disease were dealt with in Ham Green Hospital.

We have long since ceased to believe that the main function of a fever hospital is to check the spread of epidemic disease in a community. Recent methods of immunizing individuals against specific infectious diseases are much more likely to do that. The modern conception of an isolation hospital is a place where infectious patients may receive the most up-to-date treatment at the hands of persons who are skilled, through long experience, in the care of such cases. Its aim is rather the saving of life and the prevention of the crippling after-effects of infectious disease.

With its admirable record of hospital provision and its reputation as a great centre of medical education Bristol might well be expected to see that its fever hospital premises are second to none in the kingdom.

It is of much interest to me to learn that by arrangement with the Somerset County Council the needs of an adjoining portion of that county will in future be met by this institution. This is yet another instance of the enlightened use of public health powers. The unnecessary multiplication of fever hospitals is to be avoided at all costs. Small institutions are only too often expensive and inefficient, and where possible one large hospital should be made to serve the requirements of a considerable aggregation of population, whether living within the same local government area or not. I hope many more authorities will follow the lead given in this matter by Bristol and Somerset.

There is only one more subject upon which I should like to touch. Many isolation hospitals are built upon very restricted sites, where even the ground necessary for extension is difficult to find. Whoever was responsible for acquiring this large estate as a hospital area for this city must have had great foresight. Not only have you ample room for all the buildings you can possibly require in the future, you have all the land you need for the production of milk and eggs and the growing of fruit and vegetables. It is just as important that sick people and persons convalescent after infectious disease should have the best possible diet as that they should be housed in good premises and looked after by a competent staff.

So far I have referred only to hospitals provided and maintained by the Bristol City Council. This city is fortunate in having two great voluntary general hospitals, which for many years have given of their best to the people of Bristol and to the promotion of medical education. I am delighted to hear that a close working arrangement is to be established between these two institutions. In future we can look forward to a real scheme of co-operation between the municipal hospitals and the voluntary hospitals of this city—each group developing freely in accordance with a well-conceived plan. This was the intention of the great Local Government Act of 1929, and I have no doubt that here, as in other ways I have indicated, Bristol will again give a lead to other local authorities throughout thc country.

